# Characterization of cuproptosis in gastric cancer and relationship with clinical and drug reactions

**DOI:** 10.3389/fcell.2023.1172895

**Published:** 2023-06-07

**Authors:** Guoming Chen, Dongqiang Luo, Xiangjun Qi, Danyun Li, Jiyuan Zheng, Yang Luo, Cheng Zhang, Qing Ren, Yuanjun Lu, Yau-Tuen Chan, Bonan Chen, Junyu Wu, Ning Wang, Yibin Feng

**Affiliations:** ^1^ School of Chinese Medicine, Li Ka Shing Faculty of Medicine, The University of Hong Kong, Pokfulam, Hong Kong SAR, China; ^2^ Guangzhou University of Chinese Medicine, Guangzhou, China; ^3^ Department of Anatomical and Cellular Pathology, State Key Laboratory of Translational Oncology, Sir Y.K. Pao Cancer Center, Prince of Wales Hospital, The Chinese University of Hong Kong, Hong Kong, China

**Keywords:** cuproptosis, GC, subtypes, CRGs, drug reaction, prognostic model

## Abstract

Gastric cancer (GC) is the fifth most common cancer worldwide. Cuproptosis is associated with cell growth and death as well as tumorigenesis. Aiming to lucubrate the potential influence of CRGs in gastric cancer, we acquired datasets of gastric cancer patients from TCGA and GEO. The identification of molecular subtypes with CRGs expression was achieved through unsupervised learning-cluster analysis. To evaluate the application value of subtypes, the K-M survival analysis was conducted to evaluate the clinical prognostic characteristics. Subsequently, we performed Gene Set Variation Analysis (GSVA) and utilized ssGSEA to quantify the extent of immune infiltration. Further, the K-M survival analysis was used to identify the prognosis-related CRGs. Next, signature genes of diagnostic predictive value were screened using the least absolute shrinkage and selection operator (LASSO) algorithm from the expression matrix for TCGA, as well as the signature gene-related subtype was clustered by the “ConsensusClusterPlus” package. Finally, the immunological and drug sensitivity assessments of the signature gene-related subtypes were conducted. A total of 173 CRGs were identified, most of the CRGs undergo copy number variation in gastric cancer. Under different patient subtypes, immune cell levels differed significantly, and the subtype exhibiting high expression of the CRGs had a better prognosis. Furthermore, we selected 34 CRGs that were highly correlated with the prognosis of gastric cancer. By constructing a multivariate Cox proportional-hazards model and a hazard scoring system, we were able to categorize patients into high- and low-risk groups based on their hazard score. K-M analysis demonstrated a significant survival disadvantage in the high-risk group. Based on Lasso regression analysis, we screened 16 signature genes, a multivariate logistic regression model [cutoff: 0.149 (0.000, 0.974), AUC:0.987] and a prognosis network diagram was constructed and their prediction efficiency for gastric cancer prognostic diagnosis was well validated. According to the signature genes, the patients were separated to two signature subtypes. We found that patients with higher CRGs expression and better prognosis had lower levels of immune infiltration. Finally, according to the results of drug susceptibility analysis, docetaxel, 5-Fluorouracil, gemcitabin, and paclitaxel were found to be more sensitive to gastric cancer.

## 1 Introduction

Gastric cancer (GC) is currently the fifth most common malignancy in the world, with more than 1 million new cases per year, and is the third leading cause of cancer-related death (Thrift and El-Serag, 2020). Owing to the low specificity of symptoms such as indigestion, loss of appetite or early satiety, weight loss, and abdominal pain, which are common in GC, its early diagnosis rate is low and more than 70% of GC patients have developed to the end-stage, making it difficult to cure (Song et al., 2017; Smyth et al., 2020). This situation makes our attention on GC centered on developing prognostic-related biomarkers or predictive models that can contribute to the diagnosis and treatment of GC (Arnold et al., 2020). Studies of GC models based on ferroptosis have provided a broader perspective on the clinical practice of GC (Li et al., 2022a; Xu et al., 2022). Therefore, optimizing the early detection, treatment, and prognosis prediction of GC from the perspective of apoptosis is indispensable.

As an essential trace metal for human body, copper is a component of the active site of many enzymes with multiple roles. In normal physiological conditions, the human body can keep intracellular copper concentrations at extremely low levels through the excretion of bile, fecal and active homeostasis mechanisms working across concentration gradients (Kim et al., 2008; Linder, 2020; Ge et al., 2022). However, several studies have demonstrated the potential of copper to inhibit GC growth and induce anti-tumor activity in the treatment of GC (Xia et al., 2019; Liu et al., 2022). It is now known that excess copper concentrations in cells can trigger a distinct form of cell death that is different from apoptosis, ferroptosis, pyroptosis, and necrosis (Kahlson and Dixon, 2022). Lately, Todd Golub’s team first discovered and proposed that copper ions bind in a direct way to lipid acylated components in the tricarboxylic acid cycle, which leads to the lipid acylated protein aggregation and the consequent iron sulfide cluster loss, as this in turn causes proteotoxic stress and eventually cell death (Tsvetkov et al., 2022). Meanwhile, they observed FDX1, a key driver of copper death, as well as lipoylated proteins, were highly correlated in human tumors, and that cuproptosis is more sensitive to cell lines with high levels of lipoylated proteins (Tsvetkov et al., 2022). Therefore, it is possible that tumor cells can be killed by activating cuproptosis. Optimizing the early detection, treatment plan and prognosis model of cancer-based on cuproptosis has a broad application prospect and clinical significance (Zhang et al., 2020; Jiang et al., 2021).

However, there has not been a comprehensive GC prognostic model based on cuproptosis yet. To fill this blank field, we developed a prognostic model related to cuproptosis to explore the prognostic effect in GC patients. It will make a contribution to striving for making accurate prognosis predictions of GC patients, providing directions for their clinical medication and improving the therapeutic effect of GC.

## 2 Materials and methods

### 2.1 Data acquisition for GC

We acquired RNA-Seq, somatic mutation, along with clinical data of GC based on the TCGA (https://www.cancer.gov/) and subsequently extracted and merged the data using R 4.1.0 software. GSE84437 (based on platform GPL6947, including 443 GC tissue samples), the series of GC was obtained from the GEO (https://www.ncbi.nlm.nih.gov/geo/). The samples of TCGA and GEO were merged after removing the batch effects with sva package.

### 2.2 Patient classification

We used the ConsensusClusterPlus to make unsupervised learning-cluster analysis for patients. Using this algorithm, we stratified GC patients into distinct subtypes in accordance with the expression of CRGs, which were subsequently visualized through the principal component analysis (PCA) algorithm.

### 2.3 Clinical prognosis, immune infiltration, and function enrichment of subtypes

To analyze the application value of different subtypes, we analyzed their differences in prognostic characteristics, immune infiltration, and function enrichment. We evaluated the clinical prognostic characteristics by log-rank test and took the Kaplan-Meier (K-M) curve. Afterwards, we performed Gene Set Variation Analysis (GSVA) using the hallmark gene set (c2.cp.kegg) obtained from the Molecular Signatures Database (https://www.gsea-msigdb.org/gsea/msigdb). We then employed single-sample Gene Set Enrichment Analysis (ssGSEA) to quantify the degree of immune infiltration. The criteria for the statistical significance was *p* < 0.05.

### 2.4 Construction of cuproptosis hazard scoring system

The K–M survival analysis was used to identify the prognosis-related CRGs. A Cox proportional-hazards model was constructed with the prognosis-related genes and the risk scores were further calculated for each patient. Depended on the median value of the risk score, the samples were separated to high- and low-risk groups, and the differences survival of the groups were evaluated through K-M survival analysis. A multivariate cox proportional-hazards model was used to analyze the value of risk score and TNM stage in the prognosis of GC. *p* < 0.05 was considered statistically significant.

### 2.5 Screening of signature gene and signature gene-related cluster

To lucubrate the influence of CRGs in GC development, we randomly split the TCGA-GC dataset, of which 80% was used as the training set and 20% as the test set, as well as filtered the prognostic-related CRGs as signature genes by lasso regression. Subsequently, the GeneMANIA (http://genemania.org/) was used to discover genes with functional similarity to the signature gene. The SCNA module of the TIMER (https://cistrome.shinyapps.io/timer/) examined the effect of different copy states of the signature gene on immune infiltration in GC compared to normal tissue. Lastly, we employed cluster analysis, utilizing the ConsensusClusterPlus package, to classify the signature gene-associated clusters based on their expression profiles.

### 2.6 Correlation of signature gene-related cluster with prognosis and immune infiltration

For the purpose of further understanding the application value of the signature gene-related cluster, we analyzed its prognostic characteristics and immune infiltration level. The K–M survival analysis was purposed to identify the prognosticative characteristics of the signature gene-related cluster. Subsequently, the immune infiltration of different modules were appraised using ssGSEA and ESTIMATE.

### 2.7 Drug susceptibility analysis

To assess whether clusters predict drug sensitivity, we used the pRRophetic to appraise the IC50 and performed a comparison between signature gene-related clusters. The statistical significance was determined using a criterion of *p* < 0.05.

## 3 Results

### 3.1 Genetic and transcriptional alterations

**FIGURE 1:**
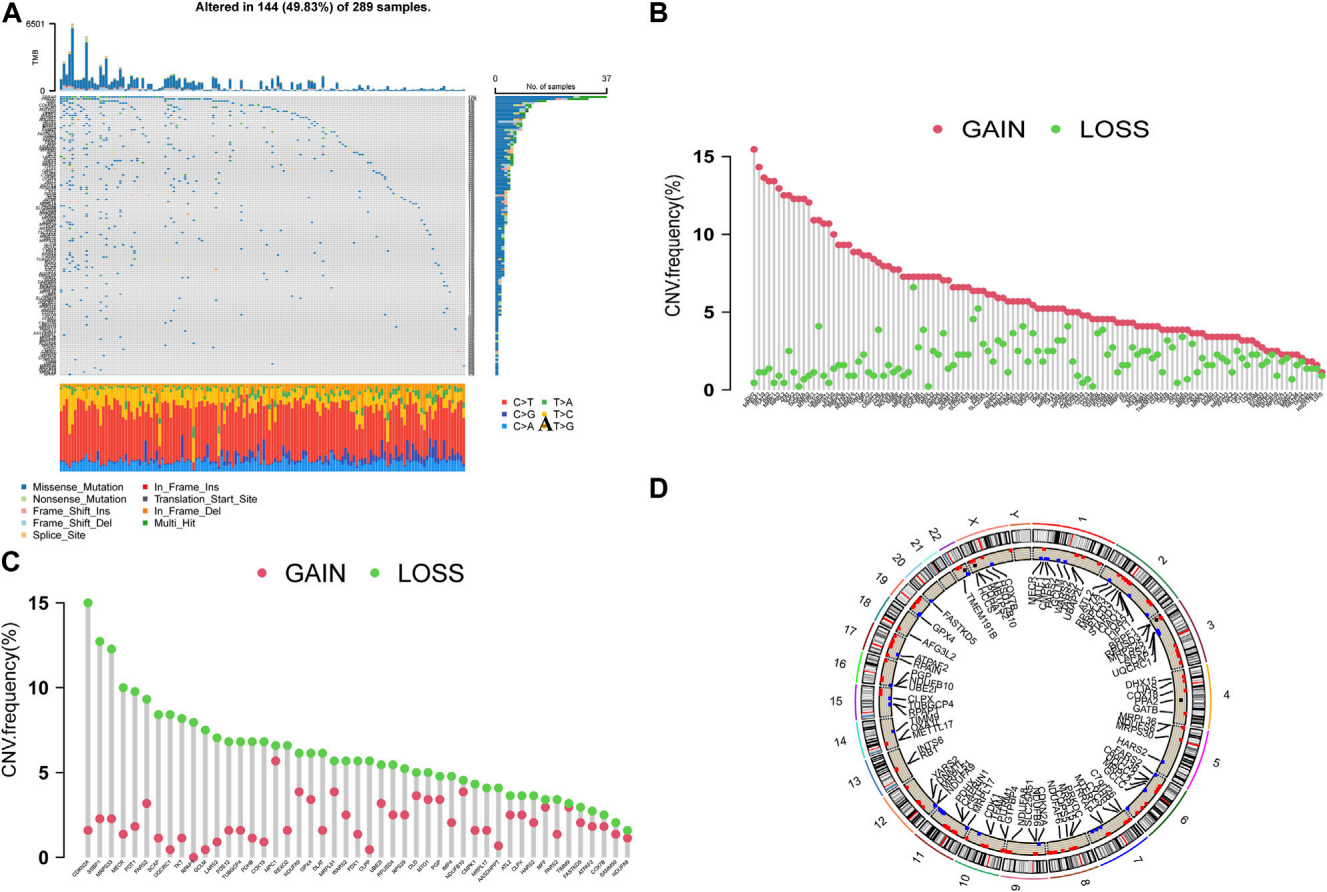
**(A)** The waterfall plot of TCGA-GC. The ordinate referred to patients, and the top of barplot referred to TMB. The numbers on the right referred to the mutation frequency for each regulator. **(B,C)** The CNV variation frequency. Green dot referred to the deletion frequency, and The red dot referred to the amplification frequency. **(D)** The positions of CNV changes in CRGs.

In the following study, we analyzed 173 CRGs. A total of 144 samples (49.83%) were mutated in the TCGA-GC cohort, with TPRAP having the highest mutation frequency, followed by PRKDC (Figure 1A). The CNV analysis showed that CNV changes were common in 173 CRGs, and 66.47% of which were concentrated in copy number amplification (Figure 1B), while copy number deletions were common in 47 CRGs cases including CDKN2A and SSBP1 (Figure 1C). Figure 1D shows the positions of CNV changes in CRGs on chromosomes.

### 3.2 Patient classification based on CRGs

**FIGURE 2:**
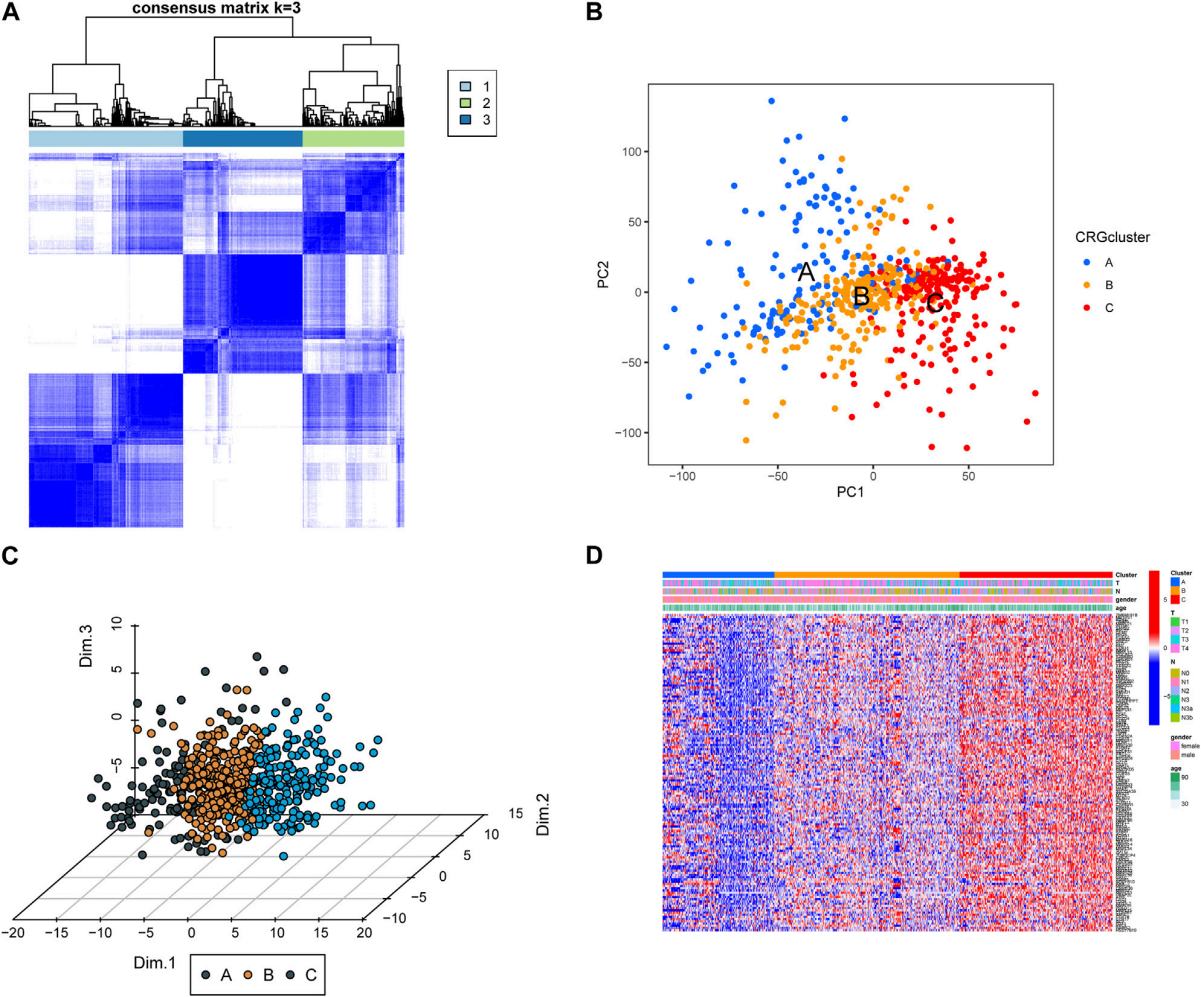
**(A)** Screening of molecular subgroups through unsupervised learning-cluster analysis. **(B,C)** Principal component analysis (PCA) of different subtypes, shows a exceptional difference in transcriptome between different classifications. **(D)** The expression level of CRGs in different CRG clusters.

To analyze the expression of CRGs in GC patients, we performed an unsupervised learning-cluster analysis in the combined gene dataset and classified the patients into CRG subtype A, CRG subtype B, and CRG subtype C (Figure 2A). The PCA analysis revealed that the different subtypes were well differentiated (Figures 2B, C). Among them, the expression level of CRGs was higher in CRG subtype C, followed by CRG subtype B, and the lowest in CRG subtype A (Figure 2D).

### 3.3 Clinical prognosis, immune infiltration, and function enrichment of different molecular subtypes

**FIGURE 3:**
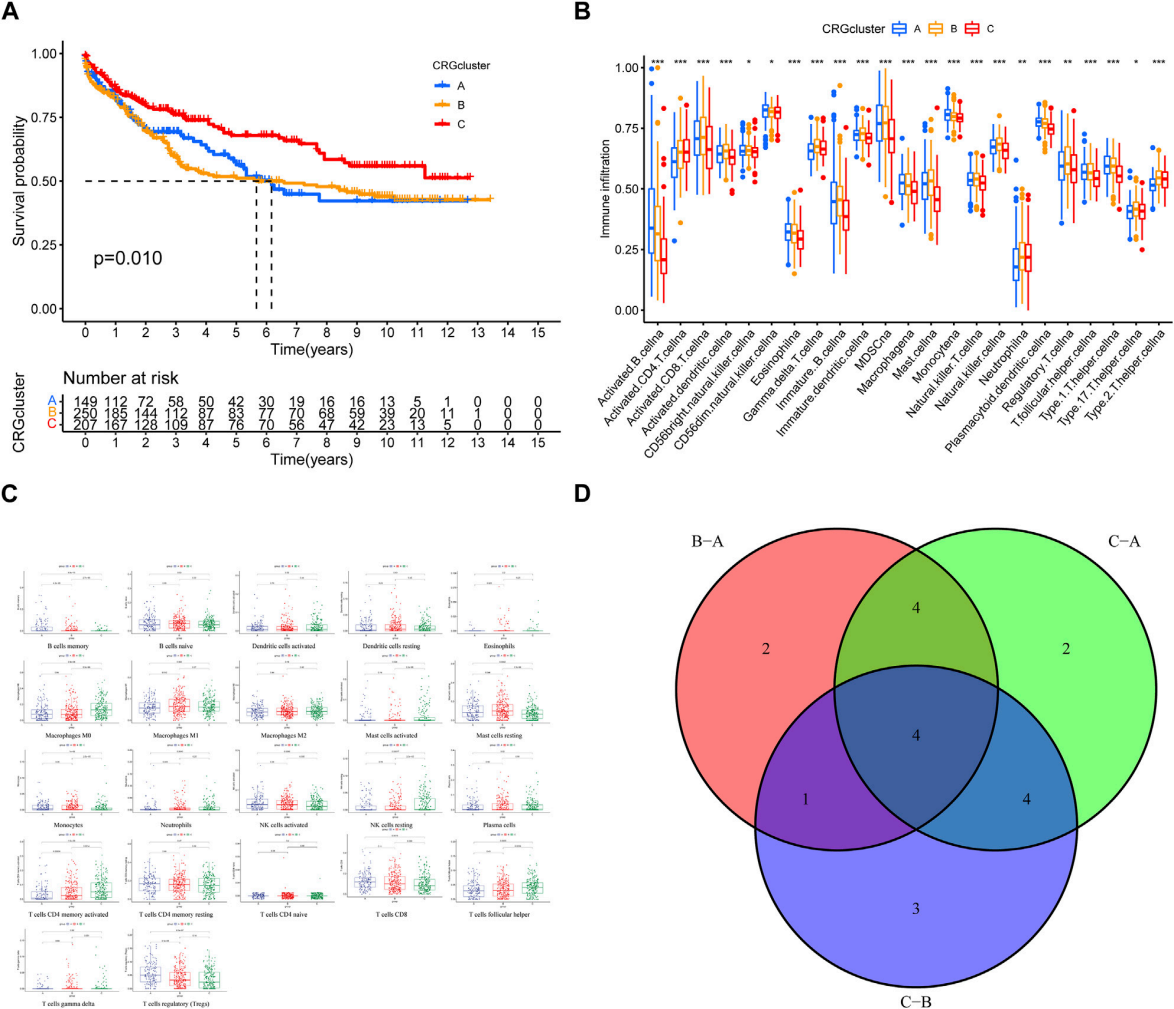
**(A)** Survival analysis for the three CRGclusters. Kaplan-Meier curves with Log-rank *p*-value 0.010 showed a significant survival difference among three clusters. **(B,C)** Immune cell infiltration in three clusters. **(D)** Venn diagram of functional distinctions among three clusters.

In order to assess the clinical difference of different subtypes, we analyzed their prognostic characteristics, immune, and functional mechanisms. Survival analysis revealed a statistically significant difference in prognosis between CRG clusters A, B, and C, with the latter exhibiting a more favorable outcome (Figure 3A). The ssGSEA analysis showed that there were significant differences in the level of immune cell infiltration among the 3 clusters (Figures 3B, C). KEGG-GSVA enrichment analysis demonstrated that the functional distinctions among the three different types of patients were mainly focused on the CALCIUM, HEDGEHOG, MAPK, and P53 signaling pathway (Figure 3D; Supplementary Table S1).

### 3.4 Hazard scoring system

**FIGURE 4:**
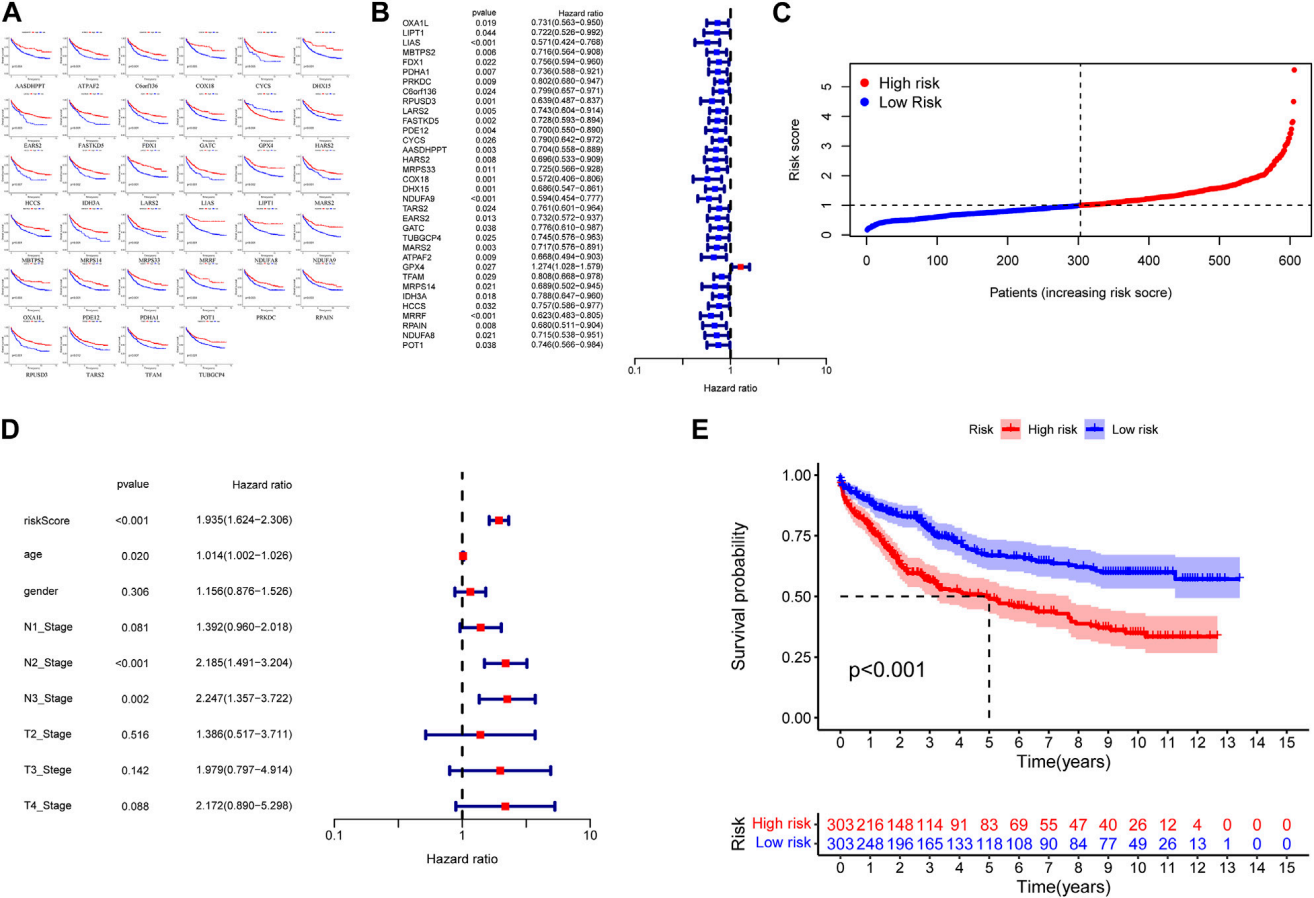
**(A)** Kaplan–Meier curves of CRGs with high correlation to prognosis. **(B)** The hazard ratio is shown as hazard ratio (95% confidence interval). **(C)** Distribution of Hazard Scores: The black dotted line represents the median hazard score cutoff, which dichotomizes patients into low-risk and high-risk groups. **(D)** The hazard ratio is shown as hazard ratio (95% confidence interval). **(E)** K‒M curves showing survival probability stratified by risk group.

To construct a hazard scoring system, we screened 173 CRGs according to their prognostic characteristics. By constructing the K–M survival analysis, we screened 34 CRGs that were highly correlated with prognosis, including AASDHPPT, ATPAF2, C6orf136, COX18, CYCS, DHX15, EARS2, FASTKD5, FDX1, GATC, GPX4, HARS2, HCCS, IDH3A, LARS2, LIAS, LIPT1, MARS2, MBTPS2, MRPS14, MRPS33, MRRF, NDUFA8, NDUFA9, OXA1L, PDE12, PDHA1, POT1, PRKDC, RPAIN, RPUSD3, TARS2, TFAM, and TUBGCP4 (Figures 4A, B). We constructed a cox proportional hazard model in accordance with the above genes and calculated the risk score, followed by a cox proportional-hazards model with the risk score and TNM as independent variables, which yield the risk score HR = 1.935 (95% CI: 1.624-2.306) with independent predictive value (Figures 4C, D). Then we performed survival analysis between the high-risk group and low-risk group, noting that the prognosis of patients in the high-risk group was worse than that those in the low-risk group (*p* < 0.001, Figure 4E).

### 3.5 Screening of signature gene and signature gene-related cluster

**FIGURE 5:**
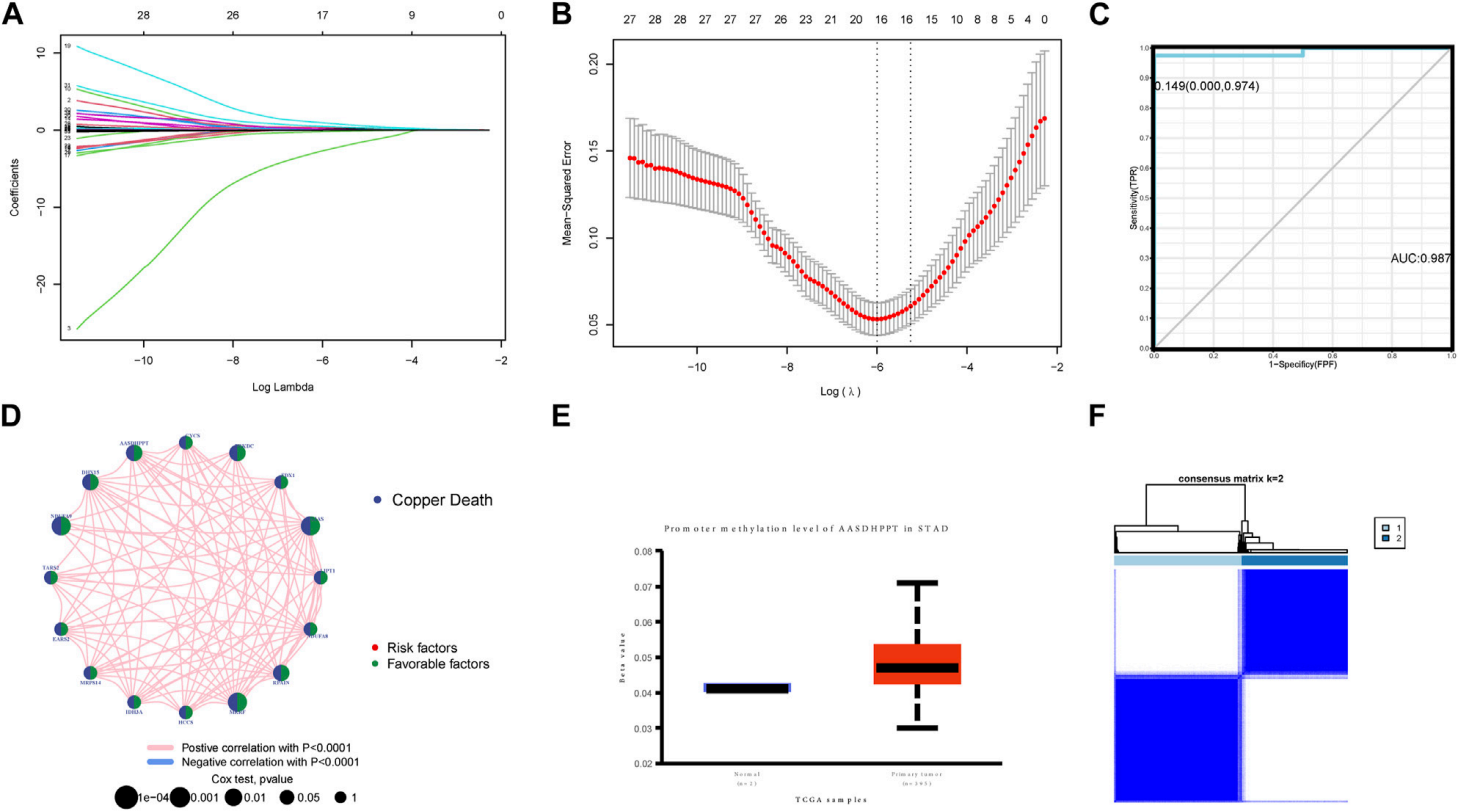
**(A,B)** Signature genes screened out by lasso analysis. **(C)** Multivariate logistic regression model of signature genes had excellent diagnostic and predictive performance (cutoff: 0.149 [0.000, 0.974], AUC: 0.987). **(D)** Prognosis network diagram. The interaction between CRGs-related regulators. The circle size showed the influence of regulator on the prognosis, and the range of values considered by log-rank test was *p* < 0.001, *p* < 0.01, and *p* < 0.05. **(E)** The consensus matrix heatmaps of consensus k-means clustering. **(F)** Consensus matrix for DNA methylation classification. This clustering is based on only 16 gene expression levels for unsupervised learning-cluster analysis.

With the aim of further screening CRGs related to prognosis, we randomly split the TCGA-GC dataset and screened the above 34 prognostic-related CRGs by lasso regression, obtaining a total of 16 signature genes (Figures 5A, B). From the construction of a multivariate logistic regression model, we found that the signature genes had excellent diagnostic and predictive performance [cutoff: 0.149 (0.000, 0.974), AUC: 0.987] (Figures 5C, D). Given the list of signature genes to query, GeneMANIA used a large amount of genomics and proteomics data to discover the 20 genes with the highest functional similarity (Supplementary Figure S2). The results of the TIMER database portray the effect of the different copy states of the 16 signature genes in GC on six types of immune infiltrating cells compared to normal tissue (Supplementary Figure S3).

Through unsupervised learning-cluster analysis, we divided the signature genes into two signature gene-related clusters, in which the CRGs expression was generally elevated in cluster B (Figures 5E, F).

### 3.6 Correlation of signature gene-related cluster with prognosis and immune infiltration

**FIGURE 6:**
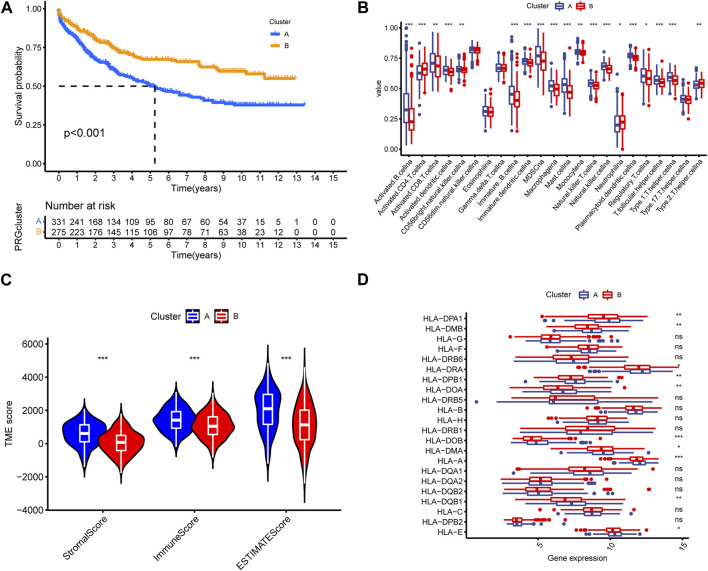
**(A)** Kaplan–Meier survival curves. The prognosis in cluster B was better than cluster A. **(B)** Cluster A was generally higher than cluster B in different immune infiltration and function. **(C,D)** TME score was utilized to differentiate the TME cluster in GC.

To evaluate the clinical application value of signature gene-related cluster, we analyzed its prognostic characteristics and immune infiltration level. Kaplan-Meier analysis demonstrated that Cluster B exhibited a more favorable prognosis than Cluster A (Figure 6A). By calculating the differences in immune microenvironment, we found that Cluster A was generally higher than Cluster B in terms of immune cell infiltration (Figure 6B). By calculating the TME score between the two types, we noticed that the Cluster A immune score was significantly better than Cluster B, and in the meantime, Cluster A has higher HLA gene expression level than Cluster B (Figures 6C, D).

### 3.7 Drug susceptibility analysis

**FIGURE 7:**
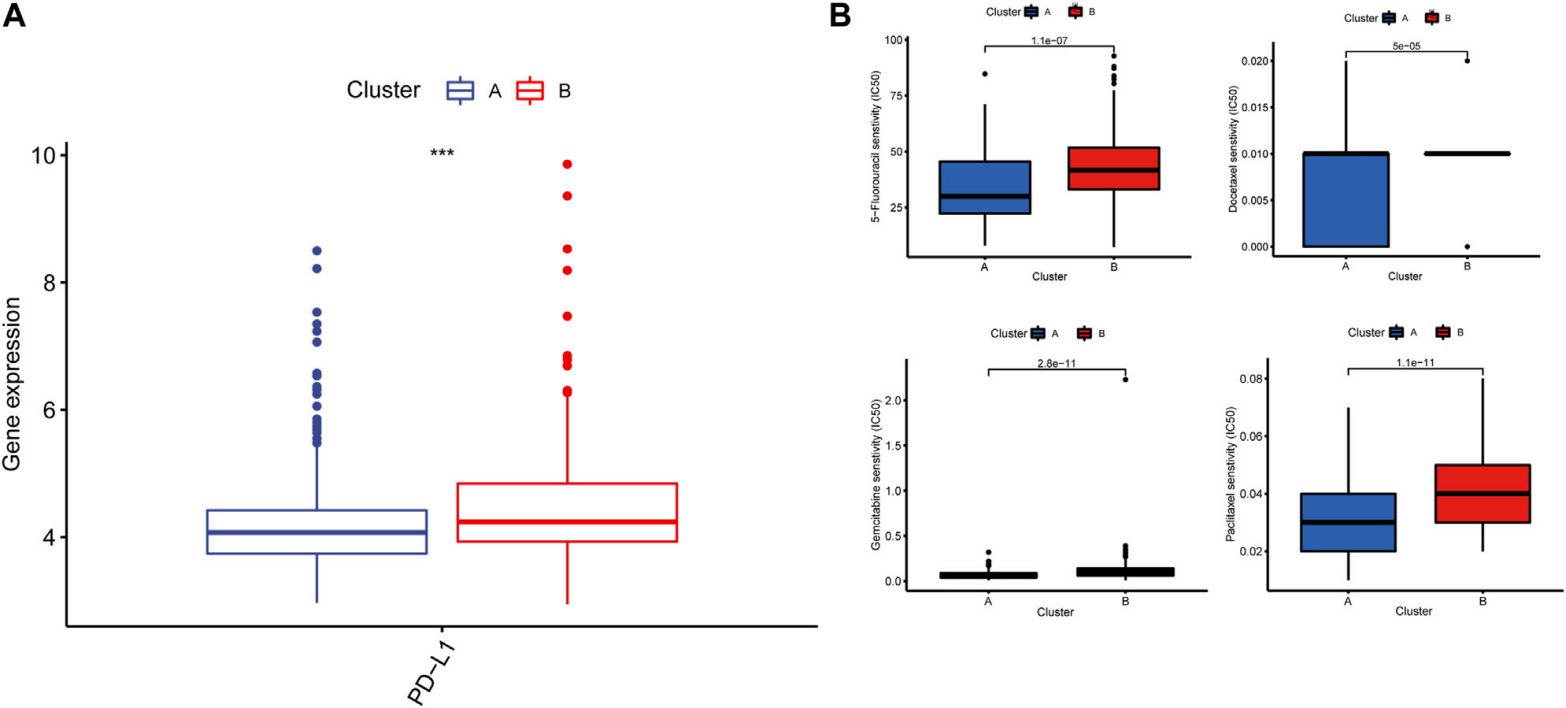
**(A)** The cluster expression of PD-L1. **(B)** The 4 GC-related therapeutic drugs.

As for the therapeutic value of the signature gene-related cluster, we compared the expression level of PD-L1 between the signature gene-related clusters where Cluster A was discovered lower than Cluster B (*p* < 0.001) (Figure 7A); Meanwhile, we appraised the drug sensitivity among signature gene-related clusters and screened the 4 GC-related therapeutic drugs, namely: docetaxel, 5-Fluorouracil, gemcitabin, paclitaxel (Figure 7B).

## 4 Discussion

Copper has a tight relationship with cancer, the imbalance of copper homeostasis can lead to cell death. Contrary to recognized pathways of cell death, copper has an impact on biological functions including mitochondrial respiration, glucose, and lipid metabolism, inducing oxidative stress and cytotoxicity, which ultimately leads to cell death (Wooton-Kee et al., 2020; Ge et al., 2022; Tsvetkov et al., 2022). However, the association between cuproptosis and GC is unclear.

In this study, data on gene mutations were collected from the TCGA database and 173 CRG were identified. And 173 CRGs were identified. CNV analysis shows that widespread CNV variation is common in CRGs. Then, using cluster analysis based on CRG expression levels, we discovered that immune cell numbers varied significantly between clusters. Apart from that, the functional analyses indicated that many pathways including mitogen-activated protein kinase (MAPK) signaling pathway, Calcium signaling pathway and Hedgehog signaling pathway participated in the process. Hedgehog signaling pathway has been demonstrated to have a crucial function in GC tumorigenesis (Fattahi et al., 2021). By downregulating cyclin D1 through the Hedgehog signaling pathway, it can inhibit the growth and cycle process of GC cells (Peng et al., 2019). Since the MAPK signaling pathway is considered an important bridge from extracellular signals to intracellular responses, relevant drugs can exert significant antitumor effects on GC through the MAPK signaling pathway (Shao et al., 2022). A territory-wide study demonstrated that the utilization of calcium channel blockers was correlated to a decreased chance of developing GC, which in part reflects the link between calcium signaling pathways and GC (Li et al., 2021).

To further evaluated GC patients’ prognosis, a multivariate Cox regression model based on 34 CRGs was constructed. It is split into two risk subgroups according to the median value from its risk score. A prognosis model based on 16 signature risk scores was constructed and validated. The ROC curve showed that the risk score performed well in predicting survival. Among 34 CRGs, the increased expression of DHX15, one of the DEXD/H box helicase family, activated p38-MAPK signal pathway and led to the inhibition of proliferation and metastasis in GC (Xiao et al., 2016). GPX4 could reduce intracellular reactive oxygen species (ROS), thereby inhibiting ferroptosis and promoting GC metastasis (Li et al., 2022b; Sugezawa et al., 2022). Knockdown of PRKDC, POT1-AS1, Mars2 suppressed growth of GC cells by inhibiting cell proliferation and cell cycle (Zhang et al., 2019; Gu and Chu, 2021; Jiang et al., 2022). Alternatively, the GeneMANIA database showed that the signature genes and their functionally similar genes were linked to a diverse array of biological processes such as cellular respiration, oxidoreductase complex, energy derivation by oxidation of organic compounds and mitochondrial protein complex. Our finding is consistent with the earlier study by Peter Tsvetkov et al. (Cui et al., 2021; Ge et al., 2022; Tsvetkov et al., 2022). These findings indicates that cuproptosis may affect GC by mediating mitochondrial respiration, and redox signaling.

Then, we further gained more insight into the relationship between risk scores and immune components and confirmed that CRGs might play essential roles in immune infiltration and tumor immune microenvironment in GC. Between high-risk and low-risk groups, differences in the tumor immune microenvironment were also assessed. GSEA results revealed that the “high-risk” group’s tumor microenvironment (TME) score was higher compared to that of the “low-risk” group. There is intricate crosstalk between cancer cells and immune cells in TME. Macrophages M1, Macrophages M2, and Tregs (T regulatory cells) showed higher infiltration in patients with CRGs of the low-risk subgroup, whereas Plasma cells, NK cells resting, and T follicular helper (Tfh) cells had higher infiltration in the low-risk subgroup. Tumor-associated macrophages (TAMs) have a dual effect, which can be separated into two major types: classically activated M1 with antitumor activity, as well as replacing activated M2 that supports cancer development (Gambardella et al., 2020). High infiltration level of macrophages is correlated with a poor overall survival rate of GC, which has a correlation in promoting inflammation, angiogenesis, hypoxia pathway, and avoiding immune surveillance (Zeng et al., 2019; Gambardella et al., 2020). Tregs participate in homeostatic regulation and tumor immune escape. GC cells secreted cytokines to recruit Tregs, whereas inducing CD4^+^ naïve T cells to differentiate into Tregs via TGF-β and induced immunosuppression (Kalamohan et al., 2014; Liu et al., 2019). Previous researches have shown that the high abundance of tumor-associated lymphocytes, like CD8^+^ T cell, CD4^+^ T cell, and NK cell, have a positive impact on the prognosis of GC by enhancing the antitumor response (Ma et al., 2022). Being a subset of CD4^+^ helper T cells, Follicular helper T cells promote tumor-associated lymphocyte activity, enhancing immune responses (Crotty, 2014; Binnewies et al., 2019). These findings suggested that CRGs may have a potential effect on immune cell dysfunction in GC, providing new ideas for subsequent immunotherapy.

Immune checkpoint inhibitors (ICIs) improve the prognosis of GC patients, among which PD-L1/PD-1 inhibitors have good anti-tumor immunological effectiveness (Chen et al., 2021; Chen et al., 2022). PD-L11/PD-1 inhibitors are recommended according to the PD-L1 score of GC. Pembrolizumab is commonly used as a third-line treatment option for patients with PD-L1 positive (CPS ≥1) gastric adenocarcinoma (Joshi and Badgwell, 2021; Zhang et al., 2022). The expression of PD-L1 has a positive tight correlation with multiple GC-specific molecular subtypes and is closely associated with immune cell infiltration such as CD8 cells (Gu et al., 2017; Shitara et al., 2020). In the study, the gene expression in PD-L1 was lower compared to that of low-risk group. Therefore, the prognostic model may forecast immune checkpoint expression levels and potentially directimmunotherapy decisions. Apart from that, the drug sensitivity between signature modules was calculated as well as 4 GC-related therapeutic drugs were screened. Research have shown that the addition of docetaxel is effective with few safety concerns in stage III GC patients (Yoshida et al., 2019). As a new fluorinated anti-metabolite, gemcitabine contributes to enhancing the individual anti-tumor activity of either 5-Fluorouracil or oxaliplatin (Nam et al., 2013). Paclitaxel is a chemotherapeutic agent that has been applied to treat various types of cancer, and its monotherapy can significantly ameliorate the tumor response and prognosis of GC (Lei et al., 2022). Immunotherapy and chemotherapy combined may provide precise treatment with various risk scores.

There are several limitations of this study. Firstly, this 16-gene prognostic model was built and verified using retrospective data from public databases. More prospective clinical data are needed for further clinical validation. Second, the link between CRGs and the tumor immune microenvironment and its mechanism requires further experimental examination.

In conclusion, our study established a new prognostic model consisting of 3 molecular subtypes based on CRGs. This model was shown to be independently associated with the prognosis of GC and was shown to be valuable in the tumor microenvironment and drug sensitivity, providing insights into predicting GC prognosis. Further research is still needed to investigate the potiential mechanism between CRGs and GC.

## Data Availability

The original contributions presented in the study are included in the article/Supplementary Material, further inquiries can be directed to the corresponding author.
