# Supplementary material for: Characterization of cuproptosis in gastric cancer and relationship with clinical and drug reactions
Source: Front Cell Dev Biol. 2023 Jun 7;11:1172895. doi: 10.3389/fcell.2023.1172895 (PMC10283039; doi:10.3389/fcell.2023.1172895)
Supplement: Supplementary file 1 [file Table1.DOCX]

**Supplementary Materials**

**Table 1**. The functional distinctions among three clusters based on KEGG-GSVA.

| KEGG signaling pathway | Contrast 1 | Contrast 2 | Contrast 3 |
| --- | --- | --- | --- |
| KEGG_ADIPOCYTOKINE_SIGNALING_PATHWAY | C-A | C-B |  |
| KEGG_B_CELL_RECEPTOR_SIGNALING_PATHWAY | C-A | C-B |  |
| KEGG_CALCIUM_SIGNALING_PATHWAY | B-A | C-A | C-B |
| KEGG_CHEMOKINE_SIGNALING_PATHWAY | C-A | C-B |  |
| KEGG_ERBB_SIGNALING_PATHWAY | B-A | C-A |  |
| KEGG_FC_EPSILON_RI_SIGNALING_PATHWAY | C-A |  |  |
| KEGG_GNRH_SIGNALING_PATHWAY | B-A | C-A |  |
| KEGG_HEDGEHOG_SIGNALING_PATHWAY | B-A | C-A | C-B |
| KEGG_INSULIN_SIGNALING_PATHWAY | C-A |  |  |
| KEGG_JAK_STAT_SIGNALING_PATHWAY | C-A | C-B |  |
| KEGG_MAPK_SIGNALING_PATHWAY | B-A | C-A | C-B |
| KEGG_MTOR_SIGNALING_PATHWAY | B-A | C-A |  |
| KEGG_NEUROTROPHIN_SIGNALING_PATHWAY | B-A | C-A |  |
| KEGG_NOD_LIKE_RECEPTOR_SIGNALING_PATHWAY | B-A |  |  |
| KEGG_NOTCH_SIGNALING_PATHWAY | C-B |  |  |
| KEGG_P53_SIGNALING_PATHWAY | B-A | C-A | C-B |
| KEGG_PPAR_SIGNALING_PATHWAY | C-B |  |  |
| KEGG_TGF_BETA_SIGNALING_PATHWAY | B-A | C-B |  |
| KEGG_TOLL_LIKE_RECEPTOR_SIGNALING_PATHWAY | C-B |  |  |
| KEGG_VEGF_SIGNALING_PATHWAY | B-A |  |  |


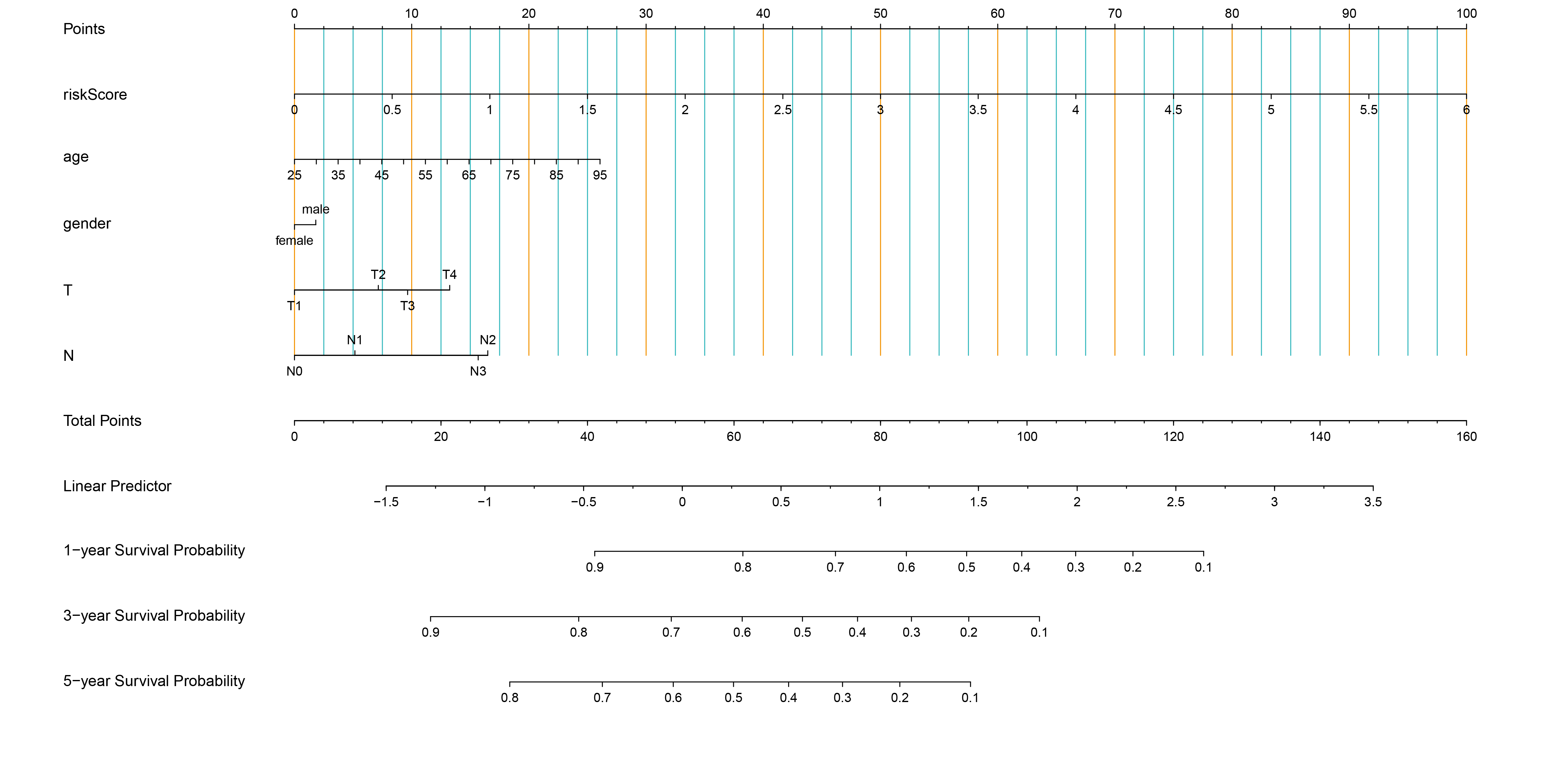


**Supplementary Figure 1.** Nomogram of hazard scoring system


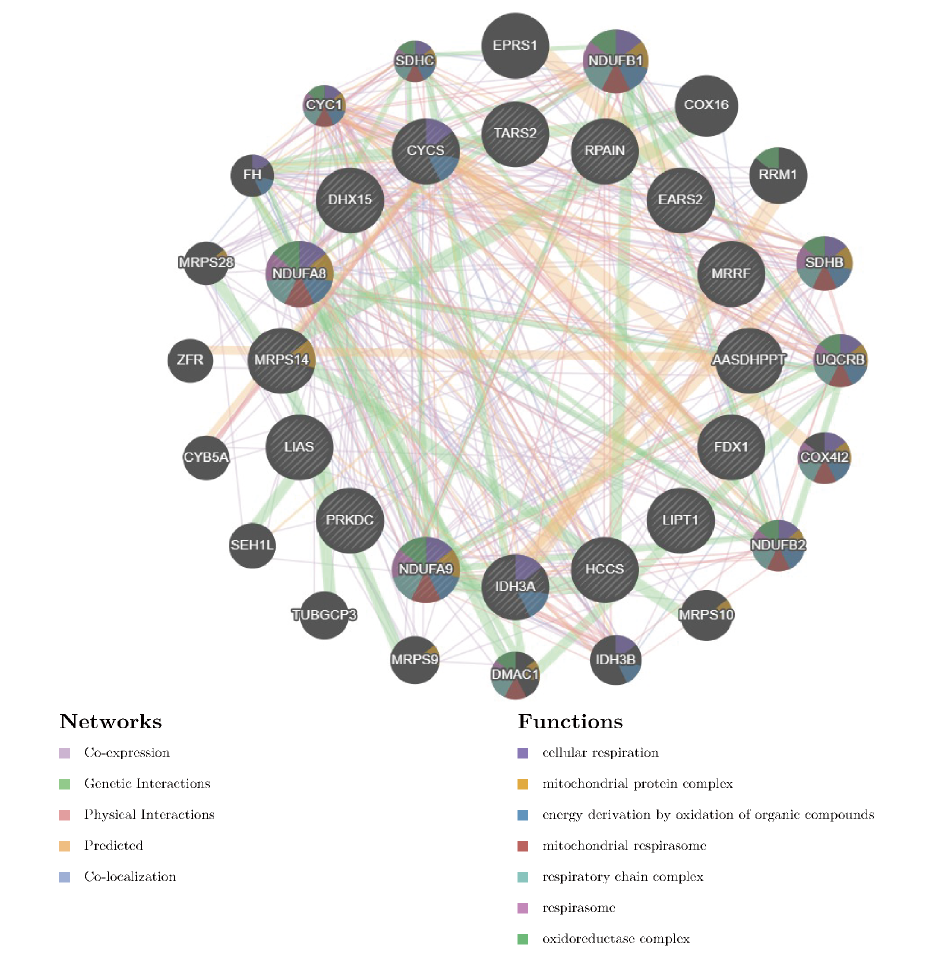


**Supplementary Figure 2.** The 20 genes with the highest functional similarity to signature genes.


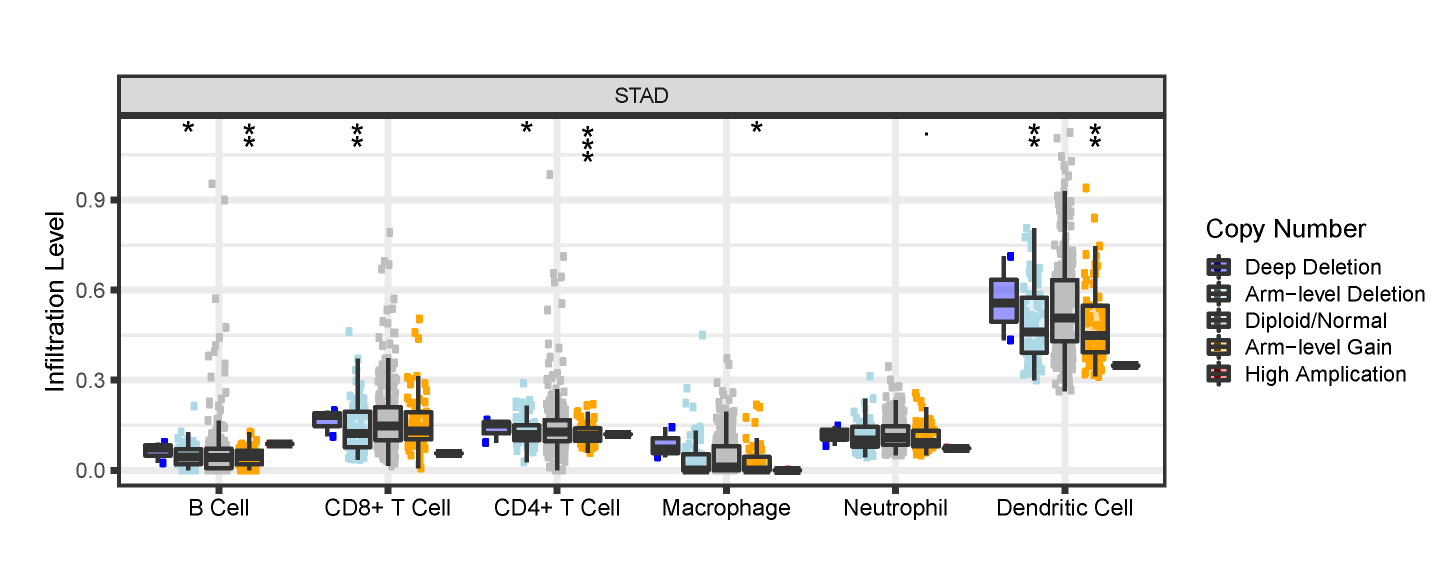


**Supplementary Figure 3.** The different copy states of the 16 signature genes in GC on six types of immune infiltrating cells.
